# Effectiveness and Safety of Fixed‐Dose Combination Metformin and Empagliflozin in Type 2 Diabetes: A Prospective, Multicenter, Real‐World Study From China

**DOI:** 10.1111/1753-0407.70273

**Published:** 2026-09-28

**Authors:** Weijian Ke, Yinghong Kong, Songbo Fu, Liyun Wang, Ying Pan, Shao Zhong, Yanhua Jiang, Hui Chen, Xuehua Jiao, Fuxing Sha, Liwu Xu, Wei Shao, Fenghua Lan, Xiaoli Chen, Bo Yan, Guangju Zhou, Ping Shi, Fen Li, Huike He, Hong Li, Guangwu Zhang, Hongyu Duan, Fang Wang, Weikun Gong, Yuxiao Bai, Bin Wang, Xin Mou, Liying He, Wukun Ma, Junhua Song, Juan Zheng, Xiaosu Bai, Xiaosi Li, Xin Yan, Yanqiu Wang, Zhenhua Wang, Juan Yuan, Wei Lu, Yuan Liang, Yufan Wang, Fenjuan Xu, Xuefeng Li, Xuan Lin, Hongbin Zhu, Lingling Xu, Xiaojia Lou, Lingxiao Wang, Lijuan Cui, Lijun Wang, Hua Qian, Yuwen Shi, Yanbing Li

**Affiliations:** ^1^ Department of Endocrinology The First Affiliated Hospital, Sun Yat‐sen University Guangzhou China; ^2^ Department of Endocrinology Changshu No.2 People's Hospital, Affiliated Changshu Hospital of Nantong University Changshu China; ^3^ Department of Endocrinology The First Hospital of Lanzhou University Lanzhou China; ^4^ Department of Endocrinology Yixing People's Hospital Yixing China; ^5^ Department of Endocrinology Affiliated Kunshan Hospital of Jiangsu University (The First People‘s Hospital of Kunshan) Suzhou China; ^6^ Department of Endocrinology Iron Coal General Hospital of Liaoning Health Industry Group Tieling China; ^7^ Department of Endocrinology The Second Hospital & Clinical Medical School, Lanzhou University Lanzhou China; ^8^ Department of Endocrinology Suzhou Ninth People's Hospital (Suzhou Ninth Hospital Affiliated to Soochow University) Suzhou China; ^9^ Department of Endocrinology Suzhou Bay Clinical College, Xuzhou Medical University Xuzhou China; ^10^ Department of Endocrinology Dengzhou People's Hospital Dengzhou China; ^11^ Department of Endocrinology The First Affiliated Hospital of Anhui University of Science and Technology (Huainan First People's Hospital) Huainan China; ^12^ Department of Endocrinology The First People's Hospital of Jiande Jiande China; ^13^ Department of Endocrinology Hangzhou Lin'an District First People's Hospital Hangzhou China; ^14^ Department of Endocrinology Zhangjiagang Hospital Affiliated to Soochow University (Zhangjiagang First People's Hospital) Zhangjiagang China; ^15^ Department of Endocrinology Mingguang People's Hospital Mingguang China; ^16^ Department of Endocrinology Affiliated Hospital of North Sichuan Medical College Nanchong China; ^17^ Department of Endocrinology Dingzhou People's Hospital Dingzhou China; ^18^ Department of Endocrinology Liuyang People's Hospital Liuyang China; ^19^ Department of Endocrinology Yiyang County People's Hospital Yiyang China; ^20^ Department of Endocrinology Zhangjiagang Hospital of Traditional Chinese Medicine Zhangjiagang China; ^21^ Department of Endocrinology Ningbo Medical Center Lihuili Hospital Ningbo China; ^22^ Department of Endocrinology Affiliated Hospital of Shaanxi University of Chinese Medicine Xianyang China; ^23^ Department of Endocrinology Wuhan Sixth Hospital (Affiliated Hospital of Jianghan University) Wuhan China; ^24^ Department of Endocrinology and Metabolism The Affiliated People's Hospital of Ningbo University Ningbo China; ^25^ Department of Endocrinology Kaifeng People's Hospital Kaifeng China; ^26^ Department of Endocrinology Beilun District People's Hospital of Ningbo Ningbo China; ^27^ Department of Endocrinology Hangzhou Red Cross Hospital (Zhejiang Hospital of Integrated Traditional Chinese and Western Medicine) Hangzhou China; ^28^ Department of Endocrinology Yiwu Central Hospital Yiwu China; ^29^ Department of Endocrinology The People's Hospital of Ruyang County Ruyang China; ^30^ Department of Endocrinology The Fourth Hospital of Changsha (Changsha Hospital of Integrated Traditional Chinese and Western Medicine) Changsha China; ^31^ Department of Endocrinology The First People's Hospital of Jiashan (Jiashan Hospital Affiliated to Jiaxing University) Jiaxing China; ^32^ Department of Endocrinology People's Hospital of Longhua Shenzhen China; ^33^ Department of Endocrinology The Second Hospital of Shijiazhuang Shijiazhuang China; ^34^ Department of Endocrinology Wuming Hospital Affiliated to Guangxi Medical University Nanning China; ^35^ Department of Endocrinology The Second Affiliated Hospital of Bengbu Medical University Bengbu China; ^36^ Department of Endocrinology Huantai County People's Hospital of Zibo Zibo China; ^37^ Department of Endocrinology, Department of Endocrinology The First People's Hospital of Guiyang Guiyang China; ^38^ Department of Endocrinology Shanghai General Hospital, Shanghai Jiao Tong University School of Medicine Shanghai China; ^39^ Department of Endocrinology The First People's Hospital of Tongxiang Tongxiang China; ^40^ Department of Endocrinology The Second Affiliated Hospital of Henan University of Science and Technology Luoyang China; ^41^ Department of Endocrinology CR & WISCO General Hospital Wuhan China; ^42^ Department of Endocrinology Dongtai People‘s Hospital Dongtai China; ^43^ Department of Endocrinology Shenzhen Hospital of Southern Medical University Shenzhen China; ^44^ Department of Endocrinology Dongyang People's Hospital Dongyang China; ^45^ Department of Endocrinology Shaoxing Central Hospital Shaoxing China; ^46^ Department of Endocrinology Benxi Steel General Hospital of Liaoning Health Industry Group Benxi China; ^47^ Department of Endocrinology First People's Hospital of Taizhou City Taizhou China; ^48^ Department of Endocrinology Taizhou Second People‘s Hospital Taizhou China

**Keywords:** China, empagliflozin, fixed‐dose combination, metformin, real‐world evidence, type 2 diabetes mellitus

## Abstract

**Background:**

This study assessed the real‐world effectiveness and safety of a fixed‐dose combination (FDC) of metformin and empagliflozin in Chinese patients with type 2 diabetes mellitus (T2DM).

**Methods:**

This prospective, multicenter, real‐world study enrolled consecutive patients with T2DM treated with the FDC of metformin and empagliflozin between August 2023 and August 2025 across 50 clinical centers in China. The primary endpoint was the 6‐month glycemic clinical control rate (HbA1c < 7.0%). The safety outcome included adverse events (AEs), major adverse cardiovascular events (MACEs), and major adverse kidney events (MAKEs).

**Results:**

A total of 2602 adults with T2DM were enrolled. The full‐analysis sets included 1719 patients, respectively. Median age was 54 years, median body mass index (BMI) was 25.7 kg/m^2^, median HbA1c was 8.3%, and median diabetes duration was 3 years; 34% of patients were female. At 6 months, the proportion of patients achieving HbA1c < 7.0% increased from 20.2% to 58.9%, with median HbA1c falling by about 1.1%–1.2%. Treatment also lowered fasting plasma glucose, body weight, and waist and hip circumference, and modestly reduced blood pressure, improving blood pressure control and shifting the BMI distribution toward normal weight. Subgroup analyses showed higher HbA1c target attainment in younger patients, those with lower baseline HbA1c, and those with higher BMI. Most patients remained on metformin/empagliflozin. AEs, MACEs, MAKEs, ketoacidosis, and genitourinary infections were infrequent.

**Conclusion:**

In this large‐scale, real‐world study in a Chinese population, the empagliflozin and metformin FDC improved long‐term HbA1c control and was well tolerated.

## Highlights

In this large‐scale real‐world Chinese cohort, the empagliflozin and metformin FDC significantly improved glycemic control, raising the proportion of patients achieving HbA1c < 7.0% from 20.2% to 58.9% at 6 months. The therapy also reduced body weight and blood pressure. Adverse events, including ketoacidosis and genitourinary infections, were infrequent, demonstrating favorable tolerability.

## Graphical abstract

This large‐scale real‐world Chinese study demonstrates glycemic clinical control rates (HbA1c > 7%) at baseline and 6 months across key subgroups, including age, baseline HbA1c, body mass index, and treatment regimen. In this prospective real‐world study of fixed‐dose combination metformin and empagliflozin in Chinese patients with type 2 diabetes, glycemic control improved significantly across all subgroups at 6 months (all *p* < 0.0001), demonstrating consistent clinical effectiveness regardless of patient characteristics.

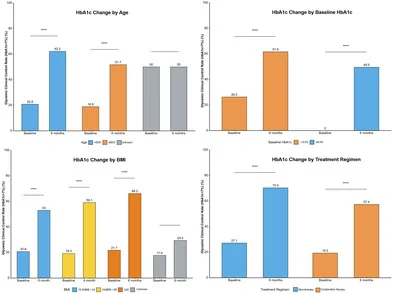

## 1 Introduction

The global diabetes epidemic represents a major challenge to public health systems worldwide [1]. Despite the ongoing introduction of new glucose‐lowering agents over the past few years, multiple unmet clinical needs persist in the management of type 2 diabetes mellitus (T2DM) in China [2]. Although traditional antidiabetic drugs (such as metformin, sulfonylureas, and insulin) are effective in reducing blood glucose, their ability to prevent cardiovascular and renal complications remains limited [3]. Consequently, the most recent national guidelines strongly recommend that T2DM patients with established atherosclerotic cardiovascular disease (ASCVD) or at high cardiovascular risk should receive glucagon‐like peptide‐1 receptor agonists (GLP‐1 RAs) or sodium‐glucose cotransporter‐2 (SGLT2) inhibitors with proven cardiovascular benefits, in combination with metformin when no contraindications exist, regardless of whether glycated hemoglobin (HbA1c) targets have been achieved [4, 5]. However, multidrug regimens can reduce adherence, leading to suboptimal glycemic control [6, 7]. Meanwhile, the rising prevalence of comorbidities, such as overweight, obesity, and metabolic‐associated fatty liver disease (MAFLD), among patients with T2DM [8, 9, 10] underscores an urgent clinical need for therapies that integrate glucose‐lowering effects with broader metabolic benefits. In this regard, fixed‐dose combination (FDC) therapies, single‐pill formulations combining traditional glucose‐lowering agents with novel drugs such as SGLT2 inhibitors or dipeptidyl peptidase‐4 (DPP‐4) inhibitors, have emerged as a promising strategy to enhance treatment adherence, therapeutic effectiveness, and the management of comorbid metabolic conditions [11, 12, 13, 14].

Metformin Hydrochloride and Empagliflozin Tablets is an FDC formulation of empagliflozin and metformin, two agents with complementary mechanisms of action. Metformin, a well‐established insulin sensitizer, lowers blood glucose primarily by suppressing hepatic glucose production, inhibiting intestinal glucose absorption, and enhancing peripheral glucose uptake and utilization [15]. Empagliflozin, a highly selective SGLT2 inhibitor, exerts its glucose‐lowering effect by blocking SGLT2 activity in the renal proximal tubules, thereby reducing glucose reabsorption and increasing urinary glucose excretion [16, 17]. In addition to its glycemic benefits, empagliflozin has demonstrated substantial cardio‐renal protective effects in patients with T2DM. The EMPA‐REG OUTCOME trial revealed that empagliflozin significantly reduces cardiovascular mortality, hospitalization for heart failure, and progression to end‐stage kidney disease among patients with established cardiovascular disease [18]. Similarly, the EMPA‐KIDNEY trial showed a 28% reduction in the composite endpoint of kidney disease progression or cardiovascular death in patients with chronic kidney disease [19]. Combining these two agents yields dual glucose‐lowering efficacy [20] while maintaining the proven cardio‐renal benefits of empagliflozin [21] and meaningful metabolic improvements. Findings from the IMAGIN Pilot Study demonstrated that empagliflozin plus metformin improved hepatic steatosis, liver enzyme profiles, and body weight in patients with T2DM and non‐alcoholic fatty liver disease (NAFLD), outperforming metformin monotherapy across these metabolic outcomes [22]. Collectively, current evidence strongly supports the empagliflozin–metformin combination, particularly in fixed‐dose form, as a preferred first‐line or early therapeutic option for T2DM management [23].

Although Metformin Hydrochloride and Empagliflozin Tablets was approved in China, large‐scale real‐world evidence on its effectiveness and safety among the Chinese population remains limited. Therefore, this study is designed to assess the real‐world effectiveness and safety of this FDC therapy for patients with T2DM in China using a prospective, multicenter, real‐world study design. Moreover, it aims to explore variations in treatment response across patient subgroups with differing baseline characteristics, thereby generating high‐quality real‐world evidence to support clinical guideline development and inform healthcare decision‐making.

## 2 Methods

### 2.1 Study Design and Patients

This prospective, multicenter, real‐world study enrolled consecutive patients with T2DM who received treatment with the FDC tablet of metformin and empagliflozin between August 2023 and August 2025 across 50 clinical centers in China. The study was conducted in accordance with the Declaration of Helsinki and the International Council for Harmonisation (ICH) Good Clinical Practice guidelines. The study protocol was approved by the Clinical Research and Laboratory Animal Ethics Committee of The First Affiliated Hospital, Sun Yat‐sen University (approval No. 414). Written informed consent was obtained from all participants prior to enrollment. The study was prospectively registered at the Chinese Clinical Trial Register (registration No. ChiCTR2300074612).

Inclusion criteria were as follows: (1) age ≥ 18 years, irrespective of gender; (2) clinically confirmed diagnosis of T2DM based on the Guidelines for the Prevention and Treatment of T2DM in China (2024 Edition) [24]; (3) first‐time prescription of the metformin and empagliflozin FDC tablet (500:5 mg, Enshuangping, Hangzhou Zhongmei Huadong Pharmaceutical Co. Ltd., China); (4) availability of a HbA1c result obtained within 2 weeks prior to enrollment; (5) voluntary participation with written informed consent. The exclusion criteria included (1) moderate to severe renal impairment (estimated glomerular filtration rate [eGFR] < 45 mL/min/1.73 m^2^), end‐stage renal disease, or dialysis; (2) acute or chronic metabolic acidosis, including diabetic ketoacidosis; (3) known history of severe hypersensitivity to empagliflozin, metformin, or any excipient of the investigational product; (4) participation in another clinical trial within the past month; or (5) any condition deemed by the investigator to make the patient unsuitable for study participation.

### 2.2 Treatment and Follow‐Up

This study prospectively collected and analyzed case data from eligible patients following screening. The screening period occurred within 14 days prior to the prescription of the metformin and empagliflozin FDC tablet. Baseline data included demographic characteristics, current and past medical history, blood pressure, height, weight, waist and hip circumference, HbA1c, fasting plasma glucose (FPG), 2‐h postprandial plasma glucose (2 h‐PPG), fasting insulin, low‐density lipoprotein cholesterol (LDL‐C), other relevant laboratory parameters, the metformin and empagliflozin FDC tablet regimen, and concomitant medications.

The treatment period extended from the date of initial prescription to the end of continuous clinical observation (minimum duration: 6 months). Follow‐up assessments were scheduled at 3 months (±28 days) and 6 months (±28 days). At each visit, data were collected on blood pressure, body measurements (height, weight, waist, and hip circumference), laboratory indicators (HbA1c, FPG, 2 h‐PPG, fasting insulin, LDL‐C, and other tests), the metformin and empagliflozin FDC tablet regimen and treatment adherence (evaluated by tracking treatment continuation, adjustments, or interruptions at each scheduled visit), concomitant medication use, and adverse events (AEs).

### 2.3 Endpoints

The primary endpoint was the 6‐month glycemic clinical control rate, defined as the proportion of patients achieving HbA1c < 7.0% [4, 5, 25]. Secondary endpoints included: (1) change in HbA1c from baseline at 3 and 6 months; (2) glycemic clinical control rate at 3 months; (3) change in FPG from baseline at 3 and 6 months; (4) changes from baseline in body weight, body mass index (BMI), waist circumference, and hip circumference at 3 and 6 months; and (5) changes in blood pressure (BP) from baseline and BP control rate (BP < 130/80 mmHg) at 3 and 6 months.

In addition, other laboratory parameters were assessed, including 2h‐PPG, fasting insulin, homeostasis model assessment of insulin resistance (HOMA‐IR), homeostasis model assessment of β‐cell function (HOMA‐β), and changes from baseline in C‐peptide, aspartate aminotransferase (AST), alanine aminotransferase (ALT), and other indices, along with patterns of antidiabetic medication use.

The safety endpoints comprised the incidence of AEs and serious AEs (SAEs), as well as the incidence of major adverse cardiovascular events (MACEs) and major adverse kidney events (MAKEs). MACE was defined as a composite of cardiovascular death, non‐fatal myocardial infarction, or non‐fatal stroke, consistent with commonly used three‐point MACE definitions in cardiovascular outcome studies [26]. MAKE was defined as a composite outcome of at least a 40% decline in estimated glomerular filtration rate (eGFR) or doubling of serum creatinine, end‐stage kidney disease, initiation of renal replacement therapy, or death due to renal causes [27].

### 2.4 Statistical Analysis

The full analysis set (FAS) was defined as the analysis population approximating the intention‐to‐treat (ITT) principle, including all patients who received at least one dose of the study drug and had at least one post‐baseline effectiveness assessment. The per‐protocol set (PPS) comprised patients who completed at least 6 months of treatment. The safety set (SS) included all patients who received at least one dose of the study drug and had at least one safety assessment.

For the primary effectiveness endpoint, missing data were imputed using the last observation carried forward (LOCF) method, whereas missing values for secondary effectiveness and safety endpoints were not imputed. Continuous variables were summarized by number of observations, mean, standard deviation, median, minimum, maximum, interquartile range (IQR), lower quartile (Q1), and upper quartile (Q3). Categorical variables were summarized as counts and percentages. McNemar's test for paired data was used to evaluate changes from baseline in the proportions of patients achieving glycemic and blood pressure control. Changes from baseline in HbA1c, BP, FPG, body weight, BMI, waist circumference, hip circumference, and other laboratory parameters were analyzed using paired *t*‐tests, rank‐sum tests, or chi‐square tests, as appropriate to the data distribution and variable type. All statistical analyses were performed in R. Two‐sided tests were applied for hypothesis testing (unless otherwise specified), with a *p* value < 0.05 considered statistically significant, and 95% confidence intervals were reported. The primary effectiveness endpoint was further evaluated in the following prespecified subgroups: (1) age ≥ 60 years versus < 60 years; (2) baseline HbA1c < 8.5% versus ≥ 8.5%; (3) BMI categories (normal weight: 18.5 kg/m^2^ ≤ BMI < 24 kg/m^2^, overweight: 24 kg/m^2^ ≤ BMI < 28 kg/m^2^, and obesity: BMI ≥ 28 kg/m^2^); and (4) monotherapy versus combination therapy.

## 3 Results

### 3.1 Characteristics of the Patients

This study enrolled 2602 patients, as depicted in the enrollment flowchart (Figure 1). The FAS comprised 1719 patients, the PPS comprised 810 patients, and the SS comprised 2125 patients.

**FIGURE 1:**
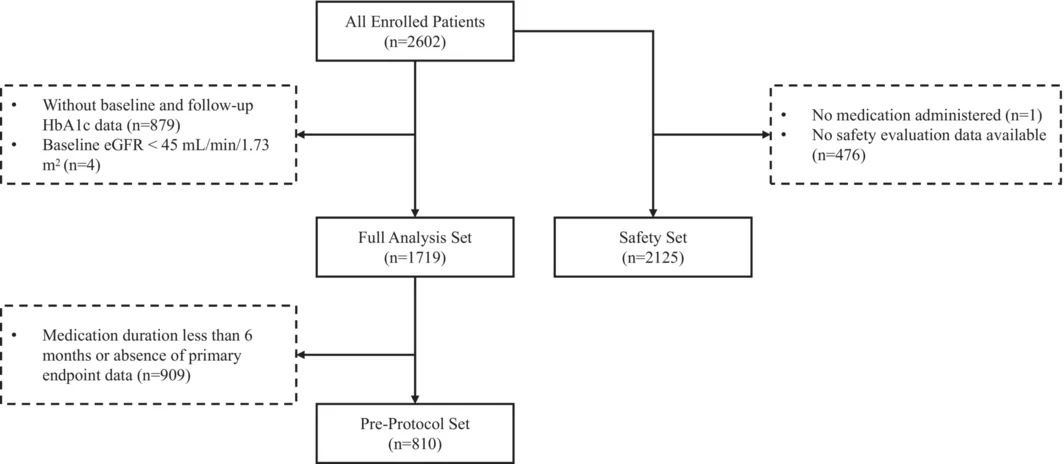
Study flowchart.

The baseline characteristics are summarized in Table 1. The median age at baseline was 54.0 years (Q1, Q3: 45.0, 61.0), mean body weight was 72.6 ± 12.8 kg, median BMI was 25.7 kg/m^2^ (23.8, 27.9), median waist circumference was 92.0 cm (86.0, 98.0), median HbA1c was 8.3% (7.1, 9.9), and median diabetes duration was 3.0 years (0.1, 8.0). A total of 584 patients (34.0%) were female. Baseline comparisons between patients receiving the metformin and empagliflozin FDC tablet monotherapy and those receiving combination therapy are shown in Table S2; patients on monotherapy were younger and had lower fasting plasma glucose and HbA1c levels than those on combination therapy, consistent with the preferential use of combination regimens in individuals with poorer baseline glycemic control.

**TABLE 1:** Demographic and baseline characteristics.

Characteristics	Total (*n* = 1719)
Age, year	54.00 (45.00, 61.00)
Sex, *n* (%)	
Male	1135 (66.03%)
Female	584 (33.97%)
Height (cm), mean ± SD	166.53 ± 8.01
Weight (kg)	71.20 (64.00, 80.00)
BMI categories, *n* (%)	
Normal (18.5 ≤ BMI < 24 kg/m^2^)	457 (26.85%)
Overweight (24 ≤ BMI < 28 kg/m^2^)	831 (48.82%)
Obesity (≥ 28 kg/m^2^)	414 (24.32%)
Waist circumference (cm)	92.00 (86.00, 98.00)
Hip circumference (cm)	98.00 (93.00, 103.00)
Waist‐to‐hip ratio	0.95 (0.91, 0.99)
Diabetes duration (years)	3.00 (0.10, 8.00)
Smoking history, *n* (%)	
Never smoked	1141 (66.38%)
Current smoker	489 (28.45%)
Former smoker	89 (5.18%)
Drinking history, *n* (%)	
Never drinker	1334 (77.60%)
Current drinker	326 (18.96%)
Former drinker	59 (3.43%)
History of CVD, *n* (%)	
Coronary heart disease	85 (7.82%)
Peripheral vascular disease	19 (1.75%)
Stroke	30 (2.76%)
Heart failure	4 (0.37%)
Laboratory parameters	
Fasting plasma glucose (mmol/L)	8.74 (7.19, 11.16)
HbA1c (%)	8.30 (7.10, 9.90)
Total cholesterol (mmol/L)	4.87 (4.06, 5.66)
Triglycerides (mmol/L)	1.84 (1.24, 2.86)
High‐density lipoprotein cholesterol (mmol/L)	1.12 (0.96, 1.33)
Low‐density lipoprotein cholesterol (mmol/L)	2.87 (2.26, 3.54)
eGFR (ml/min/1.73m^2^), mean ± SD	105.10 ± 18.08
Diastolic blood pressure (mmHg)	130.00 (120.00, 138.00)
Systolic blood pressure (mmHg)	80.00 (75.00, 87.00)
Baseline glucose‐lowering therapy, *n* (%)	
Metformin	778 (50.00%)
SGLT2i	266 (17.10%)
DPP‐4i	289 (18.57%)
AGIs	486 (31.23%)
Insulin secretagogues	442 (28.41%)
TZDs	32 (2.06%)
GLP‐1 RA	251 (16.13%)
Insulin	211 (13.56%)
Chinese patent medicine	93 (5.98%)
Other glucose‐lowering drugs	172 (11.05%)

*Note:* Data are shown as median (first quartile, third quartile), unless otherwise specified.

Abbreviations: AGIs, alpha‐glucosidase inhibitors; BMI, body mass index; CVD, cardiovascular disease; DPP‐4i, dipeptidyl peptidase‐4 inhibitor; eGFR, estimated glomerular filtration rate; GLP‐1 RA, glucagon‐like peptide‐1 receptor agonist; HbA1c, glycated hemoglobin A1c; SGLT2i, sodium‐glucose cotransporter‐2 inhibitor; TZDs, thiazolidinediones.

### 3.2 HbA1c

In the FAS, the proportion of patients achieving the HbA1c target (< 7.0%) increased from 20.2% at baseline to 58.9% at 6 months (McNemar's test, *χ* ^2^ = 570.9, *p* < 0.001), consistent with guideline‐recommended glycemic goals for most adults with T2DM. Among patients not at target at baseline, 717 achieved HbA1c < 7.0% at 6 months, whereas among those at target at baseline, only 53 failed to maintain target at 6 months. Without imputation for the primary effectiveness endpoint, among 1164 patients with evaluable effectiveness data, the HbA1c target achievement rate was 60.5% (*n* = 704). In the PPS (*n* = 810), the HbA1c target achievement rate was 61.0% (*n* = 494) (Figure 2).

**FIGURE 2:**
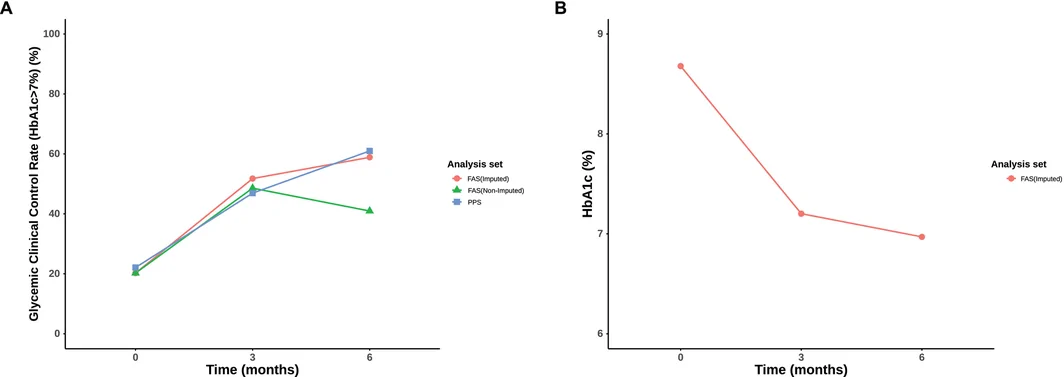
Glycemic clinical control rate in different analysis sets (the proportion of patients achieving HbA1c < 7.0%) and HbA1c change from baseline (FAS).

Among 1438 patients with 3‐month primary effectiveness data, the 3‐month HbA1c target achievement rate was 58.07%. As shown in Figure 2, median HbA1c decreased from 8.30% (7.10, 9.90) at baseline to 6.80% (6.20, 7.40) at 3 months and 6.70% (6.20, 7.40) at 6 months, corresponding to median reductions of 1.20 and 1.10 percentage points from baseline, respectively.

### 3.3 Secondary Effectiveness Indicators

Changes in FPG, body weight, waist circumference, hip circumference, waist‐to‐hip ratio, and blood pressure at the 3‐ and 6‐month follow‐up visits are summarized in Figure 3 and Table S2. After 3 months of treatment, the median FPG decreased to 6.76 mmol/L, representing a reduction of 1.70 mmol/L from baseline, and by 6 months the median further declined to 6.70 mmol/L, corresponding to a 1.82 mmol/L decrease, indicating a sustained glucose‐lowering effect over time. After 3 months, the median body weight decreased by 1.00 kg, and by 6 months the reduction reached 1.70 kg, suggesting a progressively enhanced weight‐loss effect with longer treatment duration. Waist circumference declined by a median of 1.00 cm at both 3 and 6 months; in some patients, the reduction at 6 months reached 4.00 cm, suggesting a potential decrease in visceral adiposity. In parallel with the weight‐loss pattern, hip circumference decreased by 0.50 and 1.00 cm at 3 and 6 months, respectively. The median waist‐to‐hip ratio showed no marked change from baseline, suggesting that overall fat distribution and the degree of central obesity remained essentially unchanged during the observation period and that weight loss likely reflected a relatively proportional reduction across body regions.

**FIGURE 3:**
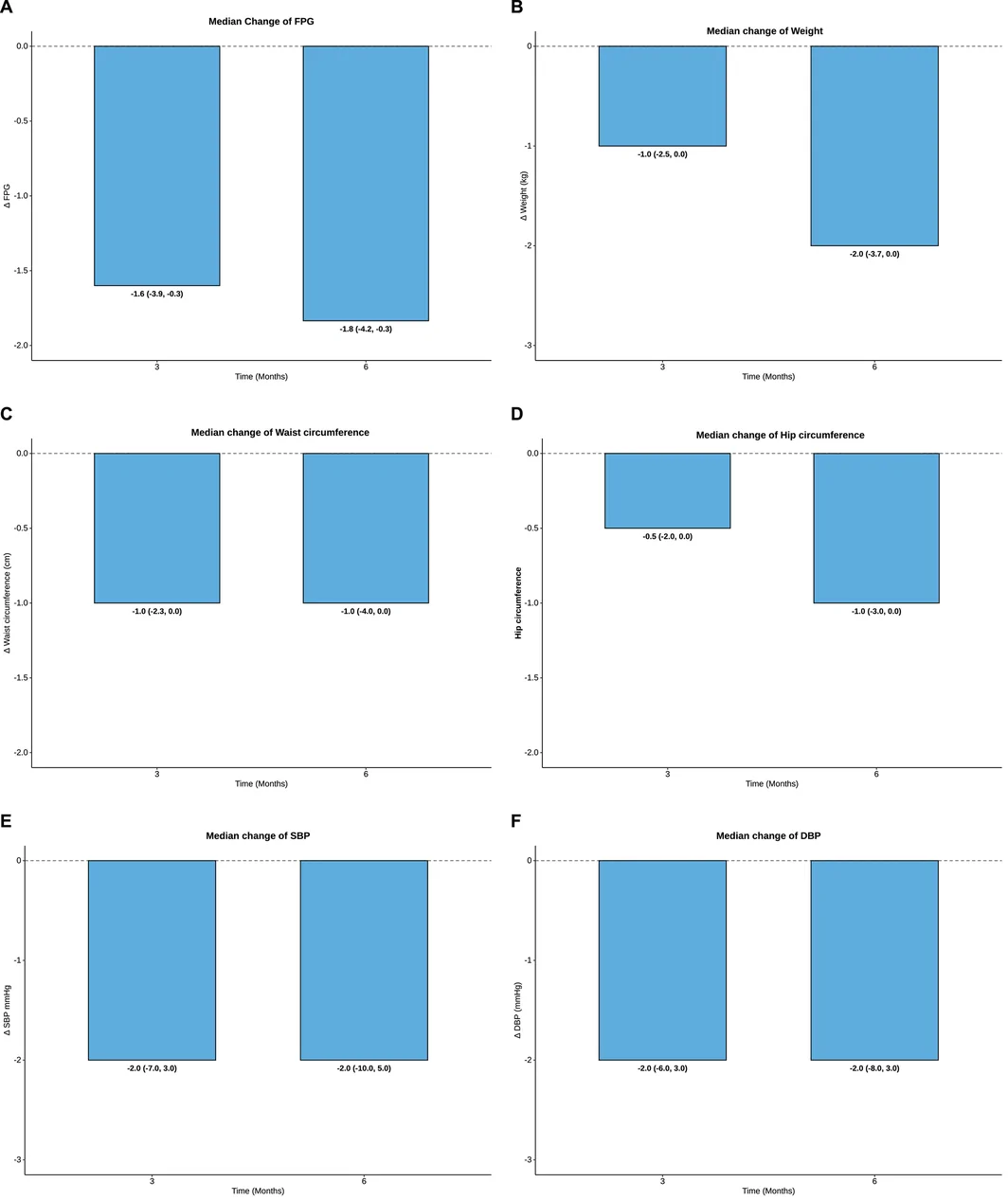
Secondary effectiveness outcomes.

Compared with baseline, the median reductions in systolic BP (SBP) and diastolic BP (DBP) after 3 months of treatment were −2.0 mmHg and −1.5 mmHg, respectively. After 6 months, the BP‐lowering effect became more evident, with median reductions of −2.0 mmHg for both SBP and DBP, and the interquartile ranges indicated that some individuals experienced greater declines (with maximum SBP reductions of up to −10.0 mmHg). The proportion of patients achieving BP control (< 130/80 mmHg) increased steadily from 30.15% at baseline to 38.90% at 6 months, corresponding to an absolute increase of 8.75% and a relative improvement of 29.0%. Relative to baseline, after 6 months of treatment, the proportion of patients with normal weight (18.5 ≤ BMI < 24 kg/m^2^) rose from 26.72% to 35.05%, whereas the proportion classified as obese (BMI ≥ 28 kg/m^2^) declined from 24.14% to 18.43%. Changes from baseline in 2h‐PPG, fasting insulin, HOMA‐IR, HOMA‐β, C‐peptide, and other laboratory parameters are detailed in Table S2.

### 3.4 Exploratory Subgroup Analyses

Analyses were performed for prespecified subgroups defined by age, baseline HbA1c, BMI category, and treatment regimen (monotherapy vs. combination therapy) (Figure 4). The 6‐month HbA1c target achievement rate was 62.2% among patients aged < 60 years and 51.7% among those aged ≥ 60 years. The corresponding rates were 61.64% for patients with baseline HbA1c < 8.5% and 49.49% for those with baseline HbA1c ≥ 8.5%, consistent with evidence that higher baseline HbA1c is associated with differential glycemic response patterns to SGLT2 inhibitor‐based therapy. By baseline BMI category, the 6‐month HbA1c target achievement rate was 52.95% in patients with normal BMI, 59.09% in those who were overweight, and 66.18% in those with obesity, aligning with prior observations that individuals with higher BMI may derive greater glycemic benefit from SGLT2 inhibitor combinations. The 6‐month HbA1c target achievement rates of patients in both the monotherapy and combination therapy groups improved compared to baseline. In the monotherapy group, the rate increased from 27.14% at baseline to 70.35%. In the combination therapy group, the rate increased from 19.34% at baseline to 57.37%.

**FIGURE 4:**
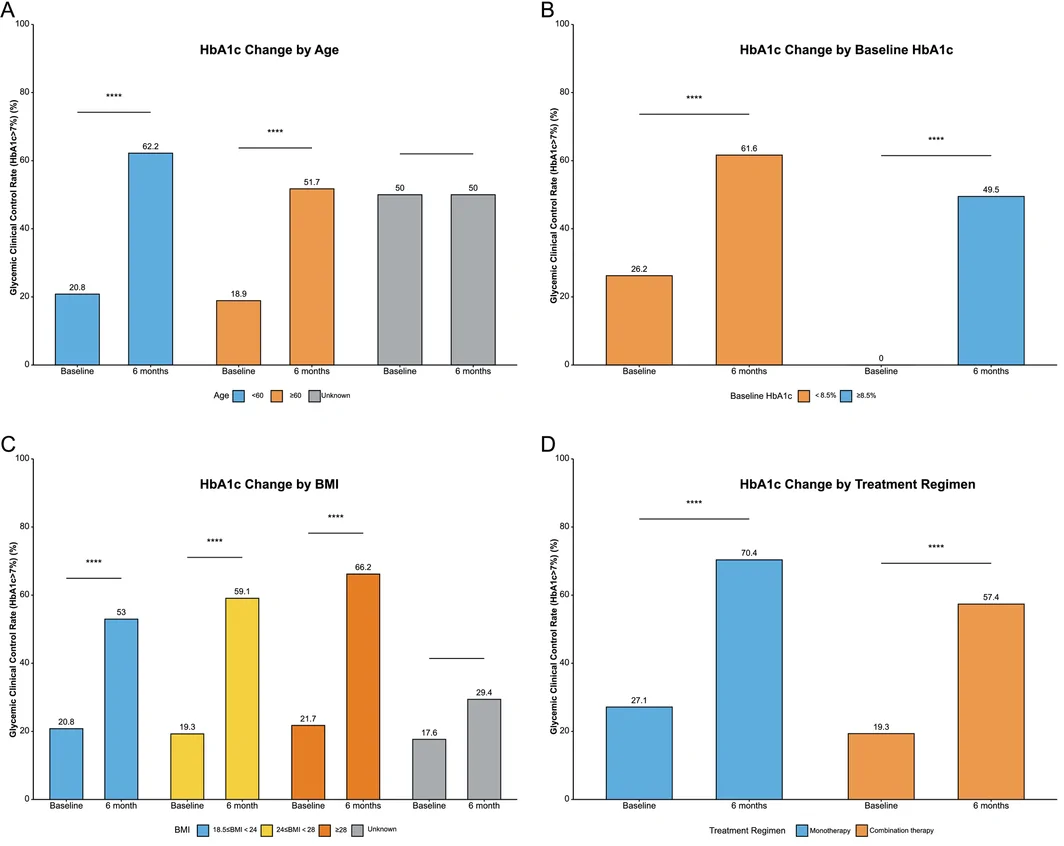
Subgroup analysis of glycemic clinical control rate (HbA1c > 7%).

### 3.5 Medication Use Pattern of Metformin and Empagliflozin FDC

During the 6‐month follow‐up, the FDC dosage regimen remained highly stable, with only 4.77% (82/1719) of patients undergoing dosage or frequency adjustments (Table S3). Medication usage during the follow‐up period is summarized in Table 2. At the 6‐month primary endpoint, 82% of patients in the FAS remained on the metformin/empagliflozin fixed‐dose combination, and 19% of these patients were receiving it concomitantly with insulin. At the 3‐month follow‐up, 92% of patients were still using the metformin/empagliflozin combination, with 24% receiving it in combination with insulin (Table 2).

**TABLE 2:** Medication usage status during the 6 months follow‐up period.

	3 months, *n* (%)	6 months, *n* (%)	Entire study period, *n* (%)
Metformin empagliflozin tablet			
*n* (missing)	1719 (0)	1719 (0)	1719 (0)
Used	1622 (94%)	1413 (82%)	1719 (100%)
Not used	97 (6%)	306 (18%)	0 (0%)
Combination with Insulin			
*n* (missing)	1622 (97)	1411 (308)	1155 (564)
Combined	384 (24%)	268 (19%)	525 (45%)
Not combined	1238 (76%)	1143 (81%)	630 (55%)
Combination with other medications			
*n* (missing)	1620 (99)	1410 (309)	1620 (99)
Combined	1173 (72%)	1004 (71%)	1485 (92%)
Not combined	447 (28%)	406 (29%)	135 (8%)

### 3.6 Safety Evaluation

A summary of AEs is shown in Table 3. In the safety set (SS), 282 patients (13.27%) experienced a total of 434 AEs. Overall, 14 patients (0.66%) reported MACEs, and two patients (0.09%) reported MAKEs. Only one patient (0.05%) developed diabetic ketoacidosis, which was managed by temporary FDC suspension, insulin therapy, and fluid resuscitation. Additionally, mild ketonuria was observed in one patient (0.05%), which required no FDC discontinuation. One patient (0.05%) experienced hypoglycemia. In addition, 12 patients (0.56%) reported urinary tract infections, and nine patients (0.42%) reported genital infections.

**TABLE 3:** Summary of AEs reported during 6‐month follow‐up in safety set.

AE, *n* (%)	Total (*N* = 2125)
Any AEs	282 (13.27%)
Any SAEs	36 (1.69)
Discontinuation due to AEs	44 (2.07%)
MACEs	14 (0.66%)
MAKEs	2 (0.09%)
Hypoglycemia	1 (0.05%)
Diabetic ketoacidosis	1 (0.05%)
Ketonuria	1 (0.05%)
Urinary tract infections	12 (0.56%)
Genital infections	9 (0.424%)

Abbreviations: AE, adverse event; MACE, major adverse cardiovascular event; MAKE, major adverse kidney event; SAE, serious adverse event.

## 4 Discussion

This prospective, multicenter, large‐scale observational real‐world study in Chinese patients with T2DM population showed that switching to the metformin and empagliflozin FDC tablet led to improvements in HbA1c, fasting glucose, body weight, waist circumference, and blood pressure. At 6 months, 58.87% of patients achieved the HbA1c target, with a median HbA1c reduction of 1.10% from baseline. Glycemic improvement was consistent across all age groups, baseline HbA1c strata, and nephropathy status. No new safety signals were observed.

HbA1c, weight, and BP responses in this real‐world cohort are broadly in line with, though numerically smaller than, those reported in randomized trials and systematic reviews of empagliflozin‐metformin therapy [20, 28, 29, 30, 31]. In this study, the 6‐month HbA1c target achievement rate approached 59%, with median HbA1c falling by about 1.1%–1.2%, which is directionally consistent with randomized controlled trials (RCTs) of empagliflozin plus metformin. Hadjadj et al. [20] reported HbA1c reductions of approximately 1.9%–2.1% over 24 weeks in 1364 drug‐naïve patients receiving initial empagliflozin‐metformin combination therapy, along with higher proportions achieving HbA1c < 7% than with either agent alone. Longer‐term add‐on studies similarly showed sustained HbA1c reductions of about 0.6%–0.8% at 76 weeks when empagliflozin was added to metformin, with greater absolute reductions in those with higher baseline HbA1c, mirroring the pattern seen in this study, where patients with baseline HbA1c < 8.5% achieved higher target rates than those with HbA1c ≥ 8.5% [30, 31]. The modest weight loss (median −1.7 kg at 6 months) and small BP reductions (median SBP/DBP −2.0/−2.0 mmHg) observed here are consistent with the direction of effect in RCTs, but somewhat smaller in magnitude. In initial combination trials, empagliflozin‐metformin twice daily reduced body weight by roughly 2.8–3.8 kg and SBP/DBP by about 2–3/2 mmHg over 24 weeks, and add‐on trials in patients on background metformin (with or without other agents) reported weight losses of 2–4 kg and SBP reductions of 3–5 mmHg over 24–76 weeks. Consistent with the present findings, systematic and narrative reviews of empagliflozin and metformin combinations conclude that this regimen improves glycemic control, promotes weight loss, and modestly reduces BP, thereby offering multidimensional cardiometabolic benefits [28, 29, 31, 32]. The attenuated absolute effect sizes in this study relative to landmark RCTs are plausibly related to differences in patient selection and treatment background. Unlike drug‐naïve or tightly defined trial populations (often restricted by baseline HbA1c, BMI, and prior therapy), participants in this real‐world cohort had already received systematic treatment before switching to the metformin/empagliflozin fixed‐dose combination, with heterogeneous disease duration, prior drug exposure, and comorbidities. Such clinical complexity may limit the observable incremental reductions in HbA1c and weight and dilute BP effects, even though the directionality of benefit is preserved and target attainment remains high, underscoring the external validity of RCT findings when translated into routine Chinese T2DM practice [20, 28, 29, 30, 31].

The subgroup findings suggest that the metformin and empagliflozin FDC tablet provides consistent glycemic benefits across clinically relevant strata, while also revealing patterns that align with known age‐ and obesity‐related pathophysiology in T2DM. Indeed, in this study, HbA1c target attainment remained favorable in both age strata, but younger patients (< 60 years) achieved a higher 6‐month HbA1c control rate than older patients (≥ 60 years), consistent with preserved β‐cell reserve and less advanced insulin resistance. Older adults with T2DM are more often characterized by impaired postprandial glucose handling, sarcopenia, frailty, and multiple comorbidities, all of which complicate treatment intensification and limit achievable HbA1c reductions [33, 34]. In addition, age‐related loss of muscle mass and function reduces glucose disposal capacity and is associated with poorer glycemic control, helping to explain why elderly patients in routine practice may show attenuated responses compared with younger counterparts despite similar exposure to SGLT2 inhibitor‐based regimens [35]. Across BMI categories, the metformin and empagliflozin FDC tablet improved HbA1c, with numerically higher target achievement in overweight and obese patients than in those with normal BMI, which is consistent with the mechanistic profile of metformin plus empagliflozin. Obese individuals typically have more severe hepatic and peripheral insulin resistance; metformin preferentially improves hepatic insulin sensitivity and can be particularly effective in insulin‐resistant phenotypes, while empagliflozin promotes glucosuria‐mediated weight loss, reduces visceral adiposity, and enhances peripheral insulin sensitivity. RCTs of empagliflozin‐metformin combinations have shown greater absolute HbA1c and weight reductions in patients with higher baseline BMI, and a recent real‐world study reported similar trends, supporting the notion of synergistic effects of this combination in overweight/obese populations that is reproduced in the present cohort [20, 31, 36, 37]. Across treatment regimen categories, monotherapy group patients achieved a higher 6‐month HbA1c control rate than combination therapy group patients. A critical factor contributing to this observation is the disparity in baseline characteristics. As shown in Table S1, patients in the monotherapy group had lower baseline HbA1c levels compared to those in the combination therapy group. It is well established that baseline HbA1c is a strong predictor of glycemic control; patients with lower baseline values are more likely to achieve the target of < 7.0% [38, 39].

The safety findings in this study are consistent with the established safety profile of the empagliflozin and metformin combination therapy, which is generally well tolerated in patients with T2DM. In this cohort, the FDC dosage remained stable, with only 4.77% of patients requiring dosage or frequency modifications during the 6‐month follow‐up, reflecting the practical feasibility of this regimen in routine clinical settings. Furthermore, the overall incidence of adverse events was low; serious cardiovascular and renal events (MACE and MAKE) were infrequent, and only one case of ketoacidosis was reported, with low rates of urinary and genital tract infections, supporting the conclusion that long‐term use of the metformin and empagliflozin FDC tablet in routine clinical practice does not reveal new safety concerns beyond those already recognized for this drug class [21, 22, 23].

This study has several important limitations that should be acknowledged. First, as an observational, single‐arm study without randomization or a concurrent control group, it cannot fully exclude confounding or establish causal effects of the metformin and empagliflozin FDC tablet on glycemic or cardiometabolic outcomes. Second, missing primary effectiveness data at 6 months, together with differential discontinuation of the metformin and empagliflozin FDC tablet before the primary endpoint, may have introduced selection bias and potentially led to over‐ or underestimation of treatment effectiveness. Third, changes in clinical outcomes may have been influenced by concomitant modifications in background therapy, lifestyle, or clinical management rather than the medication switch alone. Finally, because patients volunteered to participate and underwent prospective follow‐up, heightened awareness, increased adherence, or other behavior changes related to study participation may limit generalizability to the broader T2DM population in routine practice.

## 5 Conclusion

In this large‐scale real‐world study of Chinese adults with T2DM, the empagliflozin and metformin FDC improved long‐term HbA1c control and was generally well tolerated over 6 months of follow‐up. Beyond lowering HbA1c, treatment was associated with beneficial changes in fasting plasma glucose, body weight, waist circumference, and blood pressure, without the emergence of new safety signals, supporting the metformin and empagliflozin FDC tablet as a pragmatic option for comprehensive cardiometabolic risk management in routine practice. Looking ahead, future research should prioritize comparative effectiveness designs that include active control groups and longer follow‐up to clarify the durability of glycemic and extra‐glycemic benefits, as well as potential effects on hard cardiovascular and renal endpoints.

## Author Contributions

Yanbing Li conceptualized and designed the study. All listed authors are participating investigators. Weijian Ke, Yinghong Kong, and Songbo Fu researched data, contributed to discussion, and wrote the initial draft. Liyun Wang and Ying Pan supervised the project, contributed to discussion, and reviewed and edited the draft. All authors approved the final version of the manuscript.

## Funding

The authors have nothing to report.

## Ethics Statement

The study protocol was approved by the Clinical Research and Laboratory Animal Ethics Committee of The First Affiliated Hospital, Sun Yat‐sen University (approval No. 414).

## Consent

Written informed consent was obtained from all participants prior to enrollment.

## Conflicts of Interest

The authors declare no conflicts of interest.

## Supporting information


**Table S1:** Baseline characteristics of monotherapy and combination therapy subgroups.
**Table S2:** Secondary effectiveness outcomes.
**Table S3:** Summary of baseline prescription, treatment interruptions, and dosage adjustments of the FDC (FAS).

## Data Availability

The data that support the findings of this study are available from the corresponding author upon reasonable request.
