# Supplementary material for: Effectiveness and Safety of Fixed‐Dose Combination Metformin and Empagliflozin in Type 2 Diabetes: A Prospective, Multicenter, Real‐World Study From China
Source: J Diabetes. 2026 Sep 28;18(10):e70273. doi: 10.1111/1753-0407.70273 (PMC13617391; doi:10.1111/1753-0407.70273)
Supplement: Supplementary file 1 [file JDB-18-e70273-s001.docx]

**Supplementary material**

**Supplementary Table S1.** Baseline characteristics of monotherapy and combination therapy subgroups

| **Characteristics** | **Monotherapy**  **(N=199)** | **Combination Therapy**  **(N=1520)** |
| --- | --- | --- |
| Age | 50.00 (41.00, 57.00) | 54.00 (45.00, 62.00) |
| Sex |  |  |
| Male | 139 (69.85%) | 996 (65.53%) |
| Female | 60 (30.15%) | 524 (34.47%) |
| Height (cm) | 169.00 (163.00, 172.00) | 168.00 (160.00, 172.00) |
| Weight (kg) | 70.00 (63.00, 80.00) | 71.50 (64.00, 80.00) |
| Body mass index (kg/m^2^) | 25.40 (23.14, 27.74) | 25.72 (23.88, 27.93) |
| Waist circumference (cm) | 90.00 (85.00, 97.00) | 93.00 (86.00, 98.50) |
| Hip circumference (cm) | 97.00 (92.00, 102.00) | 98.00 (93.00, 103.00) |
| Waist-to-hip ratio | 0.95 (0.91, 0.98) | 0.95 (0.91, 0.99) |
| Diastolic blood pressure (mmHg) | 127.50 (120.00, 135.00) | 130.00 (120.00, 138.00) |
| Systolic blood pressure (mmHg) | 79.00 (73.50, 86.00) | 80.00 (75.00, 87.00) |
| Laboratory parameters |  |  |
| Fasting plasma glucose (mmol/L) | 8.30 (7.00, 9.87) | 8.86 (7.20, 11.31) |
| 2-h postprandial glucose (mmol/L) | 13.40 (9.86, 17.05) | 15.55 (12.35, 18.60) |
| HbA1c (%) | 7.80 (6.90, 9.20) | 8.40 (7.20, 9.92) |
| HOMA-IR | 4.52 (2.27, 5.73) | 3.69 (2.17, 6.14) |
| HOMA-β | 46.66 (28.40, 69.51) | 31.49 (18.17, 65.39) |
| C-peptide (ng/mL) | 0.58 (0.39, 1.05) | 0.70 (0.49, 1.03) |

Note: Data are shown as median (first Quartile, third Quartile), unless otherwise specified.

Abbreviations: HbA1c, glycated haemoglobin A1c; HOMA‑IR, homeostasis model assessment of insulin resistance, HOMA‑β, homeostasis model assessment of β‑cell function.

**Supplementary Table S2.** Secondary effectiveness outcomes.

| Outcomes | Post-treatment | Post-treatment - baseline | Test statistic | P-value |
| --- | --- | --- | --- | --- |
| Fasting blood glucose, mmol/L: 3 months |  |  | 23.905 | <0.001 |
| n (missing) | 1527 (192) | 1473 (246) |  |  |
| Median (Q1, Q3) | 6.76 (5.98, 7.80) | -1.70 (-4.05, -0.23) |  |  |
| Fasting blood glucose, mmol/L: 6 months |  |  | 22.336 | <0.001 |
| n (missing) | 1323 (396) | 1276 (443) |  |  |
| Median (Q1, Q3) | 6.70 (5.92, 7.69) | -1.82 (-4.06, -0.24) |  |  |
| Weight, kg: 3 months |  |  | 21.215 | <0.001 |
| n (missing) | 1522 (197) | 1522 (197) |  |  |
| Median (Q1, Q3) | 70.00 (63.00, 78.00) | -1.00 (-2.40, 0.00) |  |  |
| Weight, kg: 6 months |  |  | 20.507 | <0.001 |
| n (missing) | 1336 (383) | 1336 (383) |  |  |
| Median (Q1, Q3) | 70.00 (62.05, 78.00) | -1.70 (-3.65, 0.00) |  |  |
| Waist circumference, cm: 3 months |  |  | 17.685 | <0.001 |
| n (missing) | 1489(230) | 1474(245) |  |  |
| Median (Q1, Q3) | 90.00 (85.00, 97.00) | -1.00 (-2.00, 0.00) |  |  |
| Waist circumference, cm: 6 months |  |  | 16.812 | <0.001 |
| n (missing) | 1294(425) | 1278(441) |  |  |
| Median (Q1, Q3) | 90.00 (84.50, 96.00) | -1.00 (-4.00, 0.00) |  |  |
| Hip circumference, cm: 3 months |  |  | 15.314 | <0.001 |
| n (missing) | 1481 (238) | 1462 (257) |  |  |
| Median (Q1, Q3) | 97.00 (92.00, 102.00) | -0.50 (-2.00, 0.00) |  |  |
| Hip circumference, cm: 6 months |  |  | 15.532 | <0.001 |
| n (missing) | 1287(432) | 1268(451) |  |  |
| Median (Q1, Q3) | 96.00 (91.00, 101.00) | -1.00 (-3.00, 0.00) |  |  |
| Waist-to-hip ratio: 3 months |  |  | 6.453 | <0.001 |
| n (missing) | 1480(239) | 1461(258) |  |  |
| Median (Q1, Q3) | 0.94 (0.90, 0.98) | 0.00 (-0.01, 0.01) |  |  |
| Waist-to-hip ratio: 6 months |  |  | 6.265 | <0.001 |
| n (missing) | 1286(433) | 1267(452) |  |  |
| Median (Q1, Q3) | 0.94 (0.90, 0.98) | 0.00 (-0.02, 0.01) |  |  |
| Systolic blood pressure, mmHg: 3 months |  |  | 7.409 | <0.001 |
| n (missing) | 1504 (215) | 1503 (216) |  |  |
| Median (Q1, Q3) | 127.00 (120.00, 134.00) | -2.00 (-7.00, 3.00) |  |  |
| Systolic blood pressure, mmHg: 6 months |  |  | 6.945 | <0.001 |
| n (missing) | 1325 (394) | 1324 (395) |  |  |
| Median (Q1, Q3) | 127.00 (120.00, 133.00) | -2.00 (-10.00, 4.50) |  |  |
| Diastolic blood pressure, mmHg: 3 months |  |  | 8.267 | <0.001 |
| n (missing) | 1503 (216) | 1502 (217) |  |  |
| Median (Q1, Q3) | 80.00 (74.00, 85.00) | -1.50 (-6.00, 3.00) |  |  |
| Diastolic blood pressure, mmHg: 6 months |  |  | 8.742 | <0.001 |
| n (missing) | 1324 (395) | 1323 (396) |  |  |
| Median (Q1, Q3) | 79.00 (73.00, 84.00) | -2.00 (-8.00, 3.00) |  |  |
| 2-h postprandial blood glucose, mmol/L: 3 months |  |  | 7.574 | <0.001 |
| n (missing) | 144 (1575) | 43 (1676) |  |  |
| Median (Q1, Q3) | 9.05 (7.80, 10.35) | -4.80 (-9.00, -2.40) |  |  |
| 2-h postprandial blood glucose, mmol/L: 6 months |  |  | 5.830 | <0.001 |
| n (missing) | 100 (1619) | 26 (1693) |  |  |
| Median (Q1, Q3) | 9.15 (8.15, 10.15) | -5.81 (-8.90, -2.96) |  |  |
| Fasting insulin, μU/mL: 3 months |  |  | 0.007 | 0.995 |
| n (missing) | 42 (1677) | 10 (1709) |  |  |
| Median (Q1, Q3) | 11.05 (7.50, 24.36) | -0.70 (-10.50, 12.38) |  |  |
| Fasting insulin, μU/mL: 6 months |  |  | 2.056 | 0.064 |
| n (missing) | 42 (1677) | 12 (1707) |  |  |
| Median (Q1, Q3) | 9.90 (6.88, 13.32) | -2.55 (-12.13, -0.17) |  |  |
| HOMA-IR: 3 months |  |  | 1.682 | 0.093 |
| n (missing) | 42 (1677) | 10 (1709) |  |  |
| Median (Q1, Q3) | 3.14 (2.21, 6.22) | -2.50 (-18.71, 0.33) |  |  |
| HOMA-IR: 6 months |  |  | 2.824 | 0.005 |
| n (missing) | 42 (1677) | 12 (1707) |  |  |
| Median (Q1, Q3) | 2.84 (1.83, 4.05) | -1.73 (-6.08, -0.35) |  |  |
| HOMA-β: 3 months |  |  | -1.580 | 0.114 |
| n (missing) | 42 (1677) | 10 (1709) |  |  |
| Median (Q1, Q3) | 97.28 (51.48, 238.68) | 70.93 (-20.76, 346.02) |  |  |
| HOMA-β: 6 months |  |  | -1.726 | 0.084 |
| n (missing) | 42 (1677) | 12 (1707) |  |  |
| Median (Q1, Q3) | 72.09 (37.48, 106.98) | 43.09 (-16.27, 74.87) |  |  |
| C-peptide, nmol/L: 3 months |  |  | 0.711 | 0.477 |
| n (missing) | 65 (1654) | 32 (1687) |  |  |
| Median (Q1, Q3) | 0.74 (0.52, 1.07) | -0.07 (-0.26, 0.18) |  |  |
| C-peptide, nmol/L: 6 months |  |  | 0.295 | 0.768 |
| n (missing) | 55 (1664) | 21 (1698) |  |  |
| Median (Q1, Q3) | 0.72 (0.50, 1.04) | -0.01 (-0.19, 0.13) |  |  |
| AST, U/L: 3 months |  |  | 4.193 | <0.001 |
| n (missing) | 597 (1122) | 467 (1252) |  |  |
| Median (Q1, Q3) | 21.00 (17.00, 27.00) | -1.00 (-7.00, 3.00) |  |  |
| AST, U/L: 6 months |  |  | 3.829 | <0.001 |
| n (missing) | 463 (1256) | 343 (1376) |  |  |
| Median (Q1, Q3) | 20.00 (17.00, 25.00) | -1.00 (-7.00, 3.00) |  |  |
| ALT, U/L: 3 months |  |  | 6.935 | <0.001 |
| n (missing) | 597 (1122) | 468 (1251) |  |  |
| Median (Q1, Q3) | 23.00 (16.00, 32.00) | -3.00 (-14.00, 3.00) |  |  |
| ALT, U/L: 6 months |  |  | 4.687 | <0.001 |
| n (missing) | 464 (1255) | 344 (1375) |  |  |
| Median (Q1, Q3) | 22.00 (16.55, 31.70) | -2.00 (-11.00, 4.00) |  |  |
| eGFR, mL/min/1.73m²: 3 months |  |  | 6.925 | <0.001 |
| n (missing) | 563 (1156) | 421 (1298) |  |  |
| Median (Q1, Q3) | 105.06 (92.75, 115.14) | -2.52 (-7.00, 2.02) |  |  |
| eGFR, mL/min/1.73m²: 6 months |  |  | 3.577 | <0.001 |
| n (missing) | 456 (1263) | 327 (1392) |  |  |
| Median (Q1, Q3) | 104.60 (93.10, 114.00) | -1.31 (-7.09, 3.00) |  |  |
| Serum creatinine, μmol/L: 3 months |  |  | -5.829 | <0.001 |
| n (missing) | 566 (1153) | 424 (1295) |  |  |
| Median (Q1, Q3) | 69.00 (57.00, 80.00) | 3.00 (-3.70, 8.70) |  |  |
| Serum creatinine, μmol/L: 6 months |  |  | -2.966 | 0.003 |
| n (missing) | 453 (1266) | 324 (1395) |  |  |
| Median (Q1, Q3) | 67.00 (57.00, 78.80) | 1.80 (-4.41, 8.00) |  |  |
| UACR, mg/g: 3 months |  |  | 1.477 | 0.200 |
| n (missing) | 29 (1690) | 6 (1713) |  |  |
| Median (Q1, Q3) | 17.12 (8.85, 42.41) | -18.69 (-28.38, 0.76) |  |  |
| UACR, mg/g: 6 months |  |  | 0.676 | 0.499 |
| n (missing) | 43 (1676) | 7 (1712) |  |  |
| Median (Q1, Q3) | 16.00 (4.56, 46.32) | -12.17 (-15.08, 4.83) |  |  |

Abbreviations: HOMA‑IR, homeostasis model assessment of insulin resistance, HOMA‑β, homeostasis model assessment of β‑cell function; AST, aspartate aminotransferase; ALT, alanine aminotransferase; eGFR, estimated glomerular filtration rate; UACR, urine albumin-creatinine ratio.

**Supplementary Table S3.** **Summary of baseline prescription, treatment interruptions**, **and dosage adjustments of the FDC (FAS)**

| **Characteristics** | **Total patients, n (%)** |
| --- | --- |
| **Baseline FDC prescription pattern** |  |
| Daily dosage (tablets) |  |
| 0.5 | 2 (0.12) |
| 1 | 155 (9.02) |
| 2 | 1529 (88.95) |
| 3 | 19 (1.11) |
| 4 | 14 (0.81) |
| **Administration frequency** |  |
| Once daily | 165 (9.60) |
| Twice daily | 1538 (89.47) |
| Three times daily | 16 (0.93) |
| **Treatment modifications during the 6-month follow-up** |  |
| Patients with any dosage or frequency adjustment | 82 (4.77) |
| Patients with any treatment interruption | 150 (8.73) |
| Temporary interruption with subsequent resumption | 109 (6.34) |
| Permanent discontinuation | 48 (2.79) |
